# Anaerobic Pyrolysis Chamber: An atmosphere-controlled furnace for controlled-atmosphere thermal decomposition

**DOI:** 10.1016/j.ohx.2025.e00716

**Published:** 2025-10-15

**Authors:** Ryan T. Chaffer, Joseph Johnson, Matthew J. DiDomizio, Mark B. McKinnon

**Affiliations:** UL Research Institutes, Fire Safety Research Institute, Columbia, MD, United States

**Keywords:** Thermal decomposition, Char formation, Pyrolysis

## Abstract

As some materials are heated they undergo thermal decomposition, resulting in a change in the material’s physical structure and chemical composition through a process called pyrolysis. During pyrolysis, flammable vapors are driven off the material; if those vapors are released in the presence of oxygen, flaming combustion may occur. Combustion can accelerate the decomposition process, making it difficult to isolate and harvest partially decomposed material samples for subsequent analysis. Due to this difficulty, the thermo-physical changes to materials undergoing pyrolysis have not been extensively studied. A new apparatus, termed the Anaerobic Pyrolysis Chamber (APC), was designed to produce gram-scale samples of partially decomposed materials by pyrolyzing those materials under a defined heating protocol and in an inert atmosphere. The APC consists of a decomposition chamber positioned within a muffle furnace, and an instrumentation package designed to monitor and control the temperature, pressure, and oxygen concentration within the decomposition chamber. The driving purpose of the APC was to produce solid products of pyrolysis for study in other measurement apparatus. The performance of the apparatus has been characterized, and the APC has been used to support property measurement in research studies.

## Graphical abstract

**Figure:**
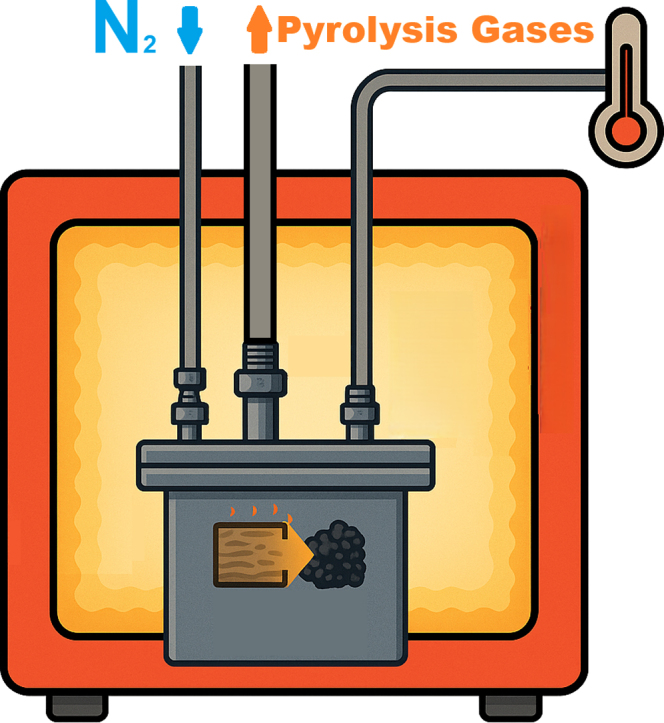

## Specifications table

**Table 1:** Specifications table.

Hardware name	Anaerobic Pyrolysis Chamber
Subject area	Engineering and material science
Hardware type	Experimental solid material preparation
Closest commercial analog	The closest commercial analog are a class of products called inert atmosphere furnaces.
Open source license	Creative Commons Attribution Share Alike 4.0 International license
Cost of hardware	$5,996.00
Source file repository	https://doi.org/10.5281/zenodo.10215409

## 1 Hardware in context

As materials become involved in fires they undergo thermal decomposition in a process called pyrolysis. As a material pyrolyzes the physical structure and chemical composition of the materials change as flammable volatiles are driven off the solid material and participate in combustion reactions to produce a flame. The physical and chemical changes to the solid materials result in changes to the thermo-physical (thermal conductivity, specific heat capacity, density) and optical (emissivity, absorption coefficient) properties [1]. An understanding of the properties of a material involved in a fire is critical to a complete understanding of the fuel generation rate of materials and the fire dynamics of a burning scenario [2].

When a material undergoes flaming combustion, fuel vapors mix with air to form a diffusion flame that is seated on the surface of the material. One side of the flame sheet is characterized by a concentration gradient of volatile flammable gases that decreases from the material surface to the flame sheet; the concentration of the gas mixture within this region is above the upper flammability limit. The opposite side of the flame sheet is characterized by ambient air and gaseous products of combustion. At the interface of these two regions is a flame sheet. Because oxygen is completely consumed in combustion reactions at the flame sheet, no oxygen diffuses through the flame and the region on the fuel-rich side of the flame may be modeled as inert.

The systems that are typically utilized to effectively simulate a burning material sustaining a diffusion flame when thermal decomposition processes are studied or measurement of the gaseous or solid products of decomposition is required involve controlled heating in a well-characterized inert atmosphere. At present there are few commercially-available products that are specifically designed for generating an inert atmosphere in a controlled high temperature environment. The commercially-available systems that are capable of generating conditions consistent with these requirements include thermal analysis instruments and inert atmosphere furnaces.

Thermal analysis techniques, including thermogravimetric analysis, differential scanning calorimetry, microscale combustion calorimetry, etc., typically utilize well-controlled heating regimes and afford the ability to adjust the gas atmosphere based on the desired measurement. These techniques and the apparatus designed to conduct such experiments are typically limited to sample masses on the order of a few milligrams, so they may only be used to investigate thermal decomposition when transport processes are not rate-limiting. Because of these limitations, the solid products of decomposition are not representative of those produced when real-scale materials are involved in fires. Additionally, the smaller scale of the material results in smaller residence times within the material matrix for the gaseous effluent which may affect the composition of gas mixture.

Commercially-available inert atmosphere furnaces are primarily used for heat treating in manufacturing processes that require inert atmosphere protection (i.e., shielding from oxidation). These furnaces are designed with various form factors and temperature ranges to provide flexibility for a wide range of applications, but, critically, these products are not designed to accommodate materials that decompose to produce flammable gases. A gas mixture that is at a high temperature and a concentration above the upper flammability limit poses a detonation or deflagration hazard if any oxygen is introduced to the mixture. Safeguards are necessary to ensure safety when decomposing materials that release flammable vapors, and this is not a primary design consideration in inert atmosphere furnaces which are not designed for such a use case.

The Anaerobic Pyrolysis Chamber (APC) was designed to be a low-cost and configurable apparatus to safely pyrolyze materials in a temperature and atmosphere controlled environment. It was designed to be compatible with any commercial muffle furnace having a suitable port opening.

## 2 Hardware description

The APC is comprised of four major components: the decomposition chamber, bubble vent, oxygen sensor module, and control module.

The decomposition chamber (DC) is used to house samples during the heating process. The DC maintains a sealed environment, free of oxygen, via continuous nitrogen injection for the duration of the heating process. The DC is positioned inside a muffle furnace, wherein samples may undergo pyrolysis during the heating process. The small form factor of the DC (5 in. by 5 in. by 2-9/32 in.) aids in its versatility to be used in a wide range of furnace sizes. The two-piece design (consisting of a base and lid that are bolted together, as opposed to a hinged design), ensures a tight seal that can withstand high pressures in the case of oxygen intrusion and a subsequent deflagration.

The DC key features are the inert gas port, thermocouple port, exhaust port, and copper sealing gasket. The 1/8 in. inert gas inlet port is used to supply nitrogen to the DC and create an anerobic environment for material decomposition to occur. A 1/16 in. ungrounded K-type Inconel thermocouple is mounted in the thermocouple port to monitor temperatures inside the DC. The DC chamber thermocouple is also used as the thermocouple sensor input for the furnace temperature controller. By incorporating a thermocouple to collect temperature internal of the DC, users are able to ensure the decomposed samples are at the minimum required temperature to produce stable, solid products of decomposition. A 1/2 in. exhaust port is used to evacuate gaseous products of decomposition and route exhaust gases to the oxygen sensor module to determine oxygen concentration inside the chamber. A copper sealing gasket is placed between the chamber base and chamber lid. With eight through-bolt fastening locations, the copper gaskets malleability allows deformation and creates a tighter seal with the stainless-steel base and lid.

**Fig. 3:**
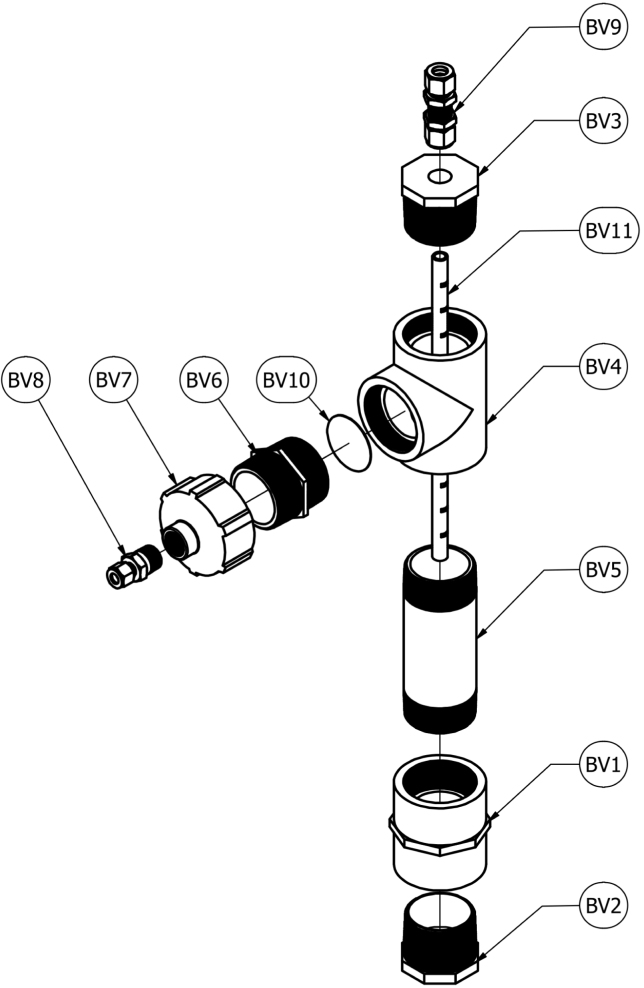
Schematic of Bubble Vent.

The bubble vent (BV), depicted in Fig. 3, is a pressure control device used to force positive pressure in the DC, cool hot exhaust gases, and filter out cooled products of decomposition. Positive pressure is ensured by restricting the exhaust gas flow, maintaining exhaust back pressure at a minimum of 5 inH_2_O (0.18 psig). Constructed from off-the-shelf components including PVC pipe, polypropylene tubing, stainless steel fittings, and mesh filters, the BV is a simplistic, easy to manufacture, and effective device to promote positive pressure. The main reservoir of the BV is constructed of 2 in. clear PVC pipe. Exhaust gases from the DC are transported to the BV inlet via 1/2 in. stainless steel tubing. A 30 ×  30 stainless steel mesh filter is included in the BV exhaust outlet to aid in removal of cooled gas condensate before the gases are transported to the oxygen sensor module. With repeated sample decompositions, the BV will require periodic maintenance of replacing the water and cleaning of the stainless steel mesh filter. The maintenance schedule will be dependent on the quantity and material type being pyrolyzed.

**Fig. 5:**
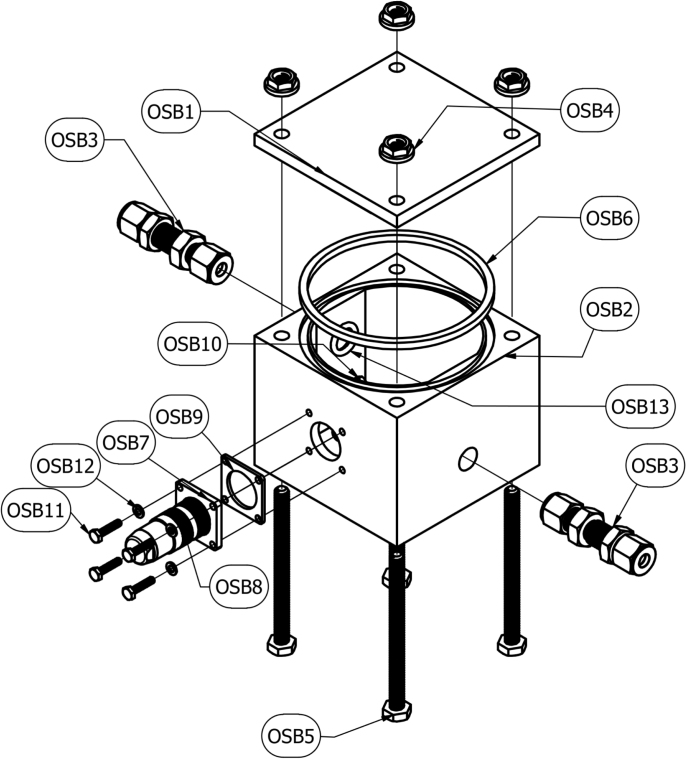
Schematic of Oxygen Sensor Module.

The oxygen sensor module (OSB), depicted in Fig. 5, is a sealed chamber equipped with an electrochemical oxygen sensor and an environmental sensor to measure the temperature, relative humidity, and pressure within the instrument. The purpose of the OS is to measure the oxygen concentration of the exhaust stream after it exits the DC and travels through the BV. 3D printed from Accura ClearVue filament and seal by an o-ring, the two-piece design allows operators to replace the electrochemical oxygen sensor when it reaches the end of its usable life. Additionally, operators can quickly identify condensate build up, if any, within the oxygen sensor module. Utilizing sealed electrical connections, power is delivered and data signals are transferred to and from the Arduino while maintaining an airtight seal. The OSB is the primary component used to ensure no leakage is present during the decomposition process, certifying an anaerobic environment within the DC.

**Fig. 8:**
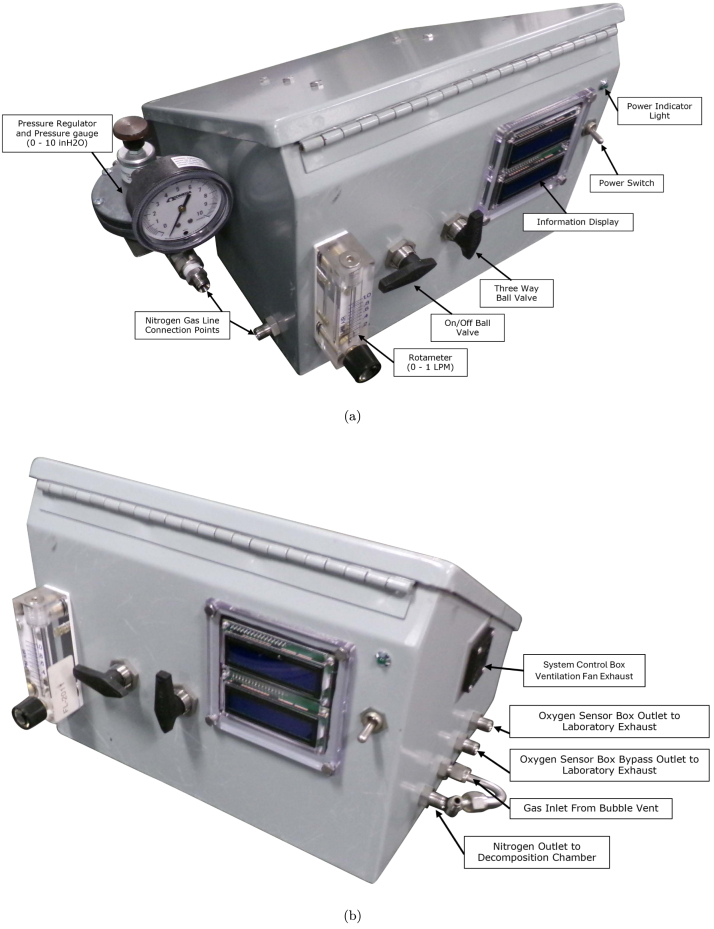
System control module exterior.

**Fig. 9:**
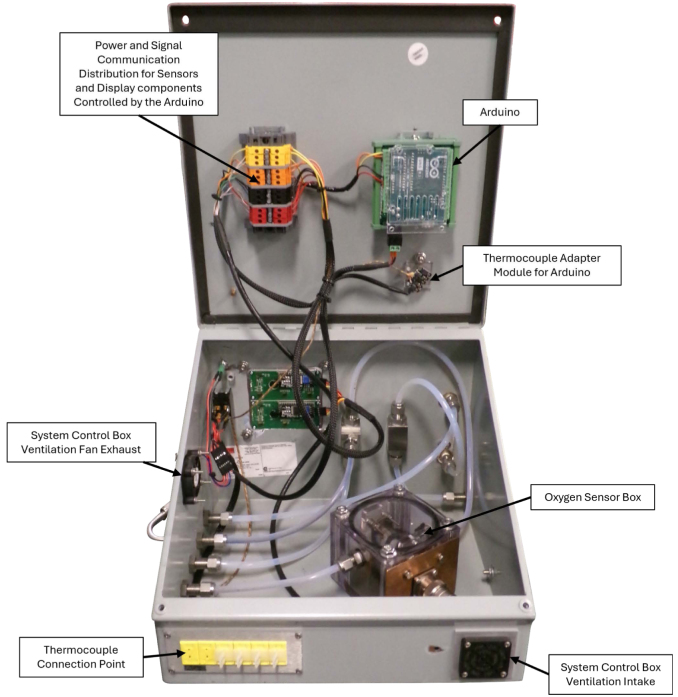
System control module interior.

The system control module (SC), seen in Fig. 8, Fig. 9, is used to control and monitor all electrical and gas flow components of the Anerobic Pyrolysis Chamber. Nitrogen is supplied to the SC via a gas bottle regulated to approximately 15 psig. The SC contains a secondary regulator to reduce the nitrogen supply pressure to 8 inH_2_O. The volume flow rate of nitrogen is controlled by a 0-1 L/min rotameter with a needle valve. The SC contains two ball valves: the first valve is used to turn the nitrogen supply to the DC on and off, while the second valve is used to divert the exhaust gas from the BV to one of two locations: the OS (for monitoring of the DC gas environment) or to laboratory exhaust (to bypass the OS and directly exhaust gases). The SC contains an Arduino Uno, which is used to collect and print measurements to the built-in displays. The displays on the SC provide an instantaneous readout of the DC temperature as well as the temperature, relative humidity, pressure, and oxygen concentration measured in the OS.

Program files to monitor and log the APC measurements are available in the APC Zenodo repository [3]. The Furnace_Data_ Collection.ino file is an Arduino sketch program (C++) that sets up the Arduino Uno to collect APC atmospheric condition data and display it on the LCD screens of the SC. The Furnace_Temp_Data_Log.py file is a Python script that is used to log the APC atmospheric condition data. When the Arduino Uno is connected to a computer communication port, the python script will intercept and connect to the Arduino. When the Python script is run, data logging will begin and will continue until the Python script is forced, by the user, to stop running. The Python script will output a .csv file containing all measurements. The Python script is not necessary for the Arduino to collect data, it is only needed for data logging, if data logging is desired by the user.

The highlights of this hardware are summarized as follows:

• Enables well-defined temperature and gas composition control in a safe and low-cost implementation for heat treatment or reactive studies.
• Allows for controlled thermal decomposition of gram-scale material samples when the solid products of decomposition must be studied.
• Provides data logging abilities to accurately report temperature conditions post sample decomposition.

## 3 Design files summary

All design files for the Anaerobic Pyrolysis Chamber can be found here https://doi.org/10.5281/zenodo.10215409.

**Table 2:** Design files summary.

Design filename	File type	Open source license	Location of the file
Bubble_Vent.stp	CAD file	CC BY-SA 4.0	https://zenodo.org/records/10215409
Chamber_Gasket.stp	CAD file	CC BY-SA 4.0	https://zenodo.org/records/10215409
Decomposition_Chamber.stp	CAD file	CC BY-SA 4.0	https://zenodo.org/records/10215409
LCD_Display_Mounting.stp	CAD file	CC BY-SA 4.0	https://zenodo.org/records/10215409
Oxygen_Sensor_box.stp	CAD file	CC BY-SA 4.0	https://zenodo.org/records/10215409
Thermocouple_Amplifier_Mounting_Bracket.stp	CAD file	CC BY-SA 4.0	https://zenodo.org/records/10215409
Bubble_Vent_Schematic.pdf	Image	CC BY-SA 4.0	https://zenodo.org/records/10215409
Decomposition_Chamber_Schematic.pdf	Image	CC BY-SA 4.0	https://zenodo.org/records/10215409
Gas Flow Diagram.pdf	Image	CC BY-SA 4.0	https://zenodo.org/records/10215409
Oxygen_Sensor_Box_Schematic.pdf	Image	CC BY-SA 4.0	https://zenodo.org/records/10215409
Wiring Diagram.pdf	Image	CC BY-SA 4.0	https://zenodo.org/records/10215409

## 4 Bill of materials

**Table 3:** Bill of materials.

Designator	Component	Number	Cost per unit	Total cost	Source of materials	Material type
DC1	Chamber Base	1	$1445.71	$1445.71	Xometry	Metal
DC2	Chamber Lid	1	$547.42	$547.42	Xometry	Metal
DC3	Copper Gasket	1	$47.20	$47.20	McMaster-Carr	Metal
DC4	High Hex Nut	12	$0.08	$0.96	McMaster-Carr	Metal
DC5	Hex Head Screw	12	$0.20	$2.40	McMaster-Carr	Metal
DC6	Split Lock Washer	12	$0.08	$0.96	McMaster-Carr	Metal
DC7	Washer	12	$0.14	$1.68	McMaster-Carr	Metal
DC8	1/2${}^{\prime \prime }$ Tube OD × 1/2${}^{\prime \prime }$ NPT Male	1	$25.96	$25.96	McMaster-Carr	Metal
DC9	1/16${}^{\prime \prime }$ Tube OD × 1/8${}^{\prime \prime }$ NPT Male	1	$16.30	$16.30	Swagelok	Metal
DC10	1/8${}^{\prime \prime }$ Tube OD × 1/8${}^{\prime \prime }$ NPT Male	1	$12.55	$12.55	McMaster-Carr	Metal
OSB1	Oxygen Sensor Module Lid	1	$203.78	$203.78	Xometry	Polymer
OSB2	Oxygen Sensor Module	1	$609.64	$609.64	Xometry	Polymer
OSB3	Through-Wall Straight Connector	2	$27.04	$54.08	McMaster-Carr	Metal
OSB4	Flange Nuts	4	$0.22	$0.88	McMaster-Carr	Metal
OSB5	Hex Head Screw	4	$0.47	$1.88	McMaster-Carr	Metal
OSB6	Square-Profile Oil-Resistant O-Ring	1	$0.47	$0.47	McMaster-Carr	Polymer
OSB7	8-pin Electrical Receptacle Kit	1	$26.88	$26.88	RS	Metal
OSB8	8-pin Electrical Plug	1	$55.55	$55.55	RS	Metal
OSB9	Plug Gasket	1	(Included With OSB7)	$0.00	RS	Inorganic
OSB10	Hex Nut	4	$0.06	$0.24	McMaster-Carr	Metal
OSB11	Hex Head Screw	4	$0.06	$0.24	McMaster-Carr	Metal
OSB12	Washer	8	$0.03	$0.24	McMaster-Carr	Metal
OSB13	Oil-Resistant Gasket	2	$0.03	$0.06	McMaster-Carr	Polymer
BV1	Straight Connector	1	$17.90	$17.90	McMaster-Carr	Polymer
BV2	Plug with External 8-Point Drive	1	$8.35	$8.35	McMaster-Carr	Polymer
BV3	Plug with External 8-Point Drive, Modified	1	$8.35	$8.35	McMaster-Carr	Polymer
BV4	Tee Connector	1	$21.09	$21.09	McMaster-Carr	Polymer
BV5	NPT Threaded Pipe	1	$54.75	$54.75	McMaster-Carr	Polymer
BV6	Male NPT Connector	1	$6.16	$6.16	McMaster-Carr	Polymer
BV7	NPT Female Reducer	1	$15.84	$15.84	McMaster-Carr	Polymer
BV8	3/8${}^{\prime \prime }$ Tube OD × 1/2${}^{\prime \prime }$ NPT Male	1	$25.07	$25.07	McMaster-Carr	Metal
BV9	Through-Wall Straight Connector	1	$55.16	$55.16	McMaster-Carr	Metal
BV10	Wire Cloth Discs	1	$1.30	$1.30	McMaster-Carr	Metal
BV11	Smooth-Bore Seamless Tubing	1	$86.25	$86.25	McMaster-Carr	Metal
SC1	Slant-Top Enclosure	1	$293.85	$293.85	McMaster-Carr	Metal
SC2	Hex Drive Screw	2	$0.10	$0.20	McMaster-Carr	Metal
SC3	DIN 3 Rail	2	$3.67	$7.34	McMaster-Carr	Metal
SC4	Screws for Plastic	10	$0.28	$2.80	McMaster-Carr	Metal
SC5	Hex Nut	10	$0.04	$0.40	McMaster-Carr	Metal
SC6	Washer	10	$0.01	$0.10	McMaster-Carr	Metal
SC7	Screw	10	$0.06	$0.60	McMaster-Carr	Metal
SC8	Washer	10	$0.04	$0.40	McMaster-Carr	Metal
SC9	Hex Head Screw	10	$0.22	$2.20	McMaster-Carr	Metal
SC10	Serrated Flange Locknut	10	$0.14	$1.40	McMaster-Carr	Metal
E1	Barrel-Style DC Connector	1	$1.24	$1.24	McMaster-Carr	Metal
E2	Toggle Switch	1	$9.4	$9.40	McMaster-Carr	Metal
E3	Panel Light	1	$5.72	$5.72	McMaster-Carr	Polymer
E4	Cooling Fan	1	$19.29	$19.29	McMaster-Carr	Polymer
E5	Cooling Fan Guard	2	$2.50	$5.00	McMaster-Carr	Polymer
E6	Red Wire	1	$3.58	$3.58	McMaster-Carr	Metal
E7	Black Wire	1	$3.58	$3.58	McMaster-Carr	Metal
E8	Orange Wire, 300V AC, 22 Gauge	1	$3.58	$3.58	McMaster-Carr	Metal
E9	Yellow Wire	1	$3.58	$3.58	McMaster-Carr	Metal
E10	Blue Wire	1	$3.58	$3.58	McMaster-Carr	Metal
E11	Terminal Block Adapter	1	$34.95	$34.95	Adafruit	Polymer
E12	Terminal Block End Cover	4	$0.43	$1.72	McMaster-Carr	Polymer
E13	Red Terminal Block	3	$1.41	$4.23	McMaster-Carr	Polymer
E14	Black Terminal Block	3	$1.41	$4.23	McMaster-Carr	Polymer
E15	Yellow Terminal Block	3	$1.41	$4.23	McMaster-Carr	Polymer
E16	Orange Terminal Block	3	$1.41	$4.23	McMaster-Carr	Polymer
E17	Terminal Block End Stop	2	$0.76	$1.52	McMaster-Carr	Polymer
E18	Noninsulated Jumper	2	$7.39	$14.78	McMaster-Carr	Metal
E19	Crimp Connector Housing	1	$0.13	$0.13	Pololu	Polymer
E20	Crimp Connector Housing	1	$0.17	$0.17	Pololu	Polymer
E21	Female Crimp Pins	1	$0.08	$0.08	Pololu	Polymer
E22	Male Crimp Pins	1	$0.12	$0.12	Pololu	Polymer
E23	Cat6 Right Angle Cable	1	$6.00	$6.00	Cable Matters	Polymer
E24	Adapter Cord	1	$14.84	$14.84	McMaster-Carr	Polymer
A1	Thermocouple Amplifier	1	$14.95	$14.95	Adafruit	Polymer
A2	Oxygen Sensor	1	$58.00	$58.00	Arduino	Polymer
A3	Arduino Uno Rev3	1	$25.30	$25.30	Arduino	Polymer
A4	LCD Display Module	2	$5.30	$10.60	GeeekPi	Polymer
A5	Temperature, Humidity, Pressure Sensor	1	$14.95	$14.95	Adafruit	Polymer
A6	Male Headers	3	$0.95	$2.85	Adafruit	Polymer
A7	Expandable Sleeving	10	$2.24	$22.40	McMaster-Carr	Polymer
A8	Thermocouple Probes	1	$45.45	$45.45	Omega	Metal
A9	Thermocouple Connectors	1	$58.65	$58.65	Omega	Polymer
A10	K Type Thermocouple Wire	1	$29.92	$29.92	Omega	Metal
A11	K Type Thermocouple Extension Wire	1	$47.69	$47.69	Omega	Metal
A12	Insulated Wire Ferrules	1	$9.12	$9.12	McMaster-Carr	Metal
A13	Heat Shrink Tubing	1	$13.99	$13.99	Wirefy	Polymer
P1	10 in WC Pressure Regulator	1	$921.34	$921.34	Equilibar	Metal
P2	Tube × NPT Adapter	4	$11.03	$22.06	McMaster-Carr	Metal
P3	Through-Wall Straight Connector	5	$27.04	$135.20	McMaster-Carr	Metal
P4	Panel-Mount Valve	1	$128.38	$128.38	McMaster-Carr	Metal
P5	Panel-Mount Flow Meter	1	$124.69	$124.69	Omega	Polymer
P6	Diverting Valve	1	$177.05	$177.05	McMaster-Carr	Metal
P7	Semi-Clear Tubing	1	$30.00	$30.00	McMaster-Carr	Polymer
P8	Smooth-Bore Tubing	1	$12.32	$12.32	McMaster-Carr	Metal
P9	90 Degree Elbow Reducer	1	$57.97	$57.97	McMaster-Carr	Metal
P10	Smooth-Bore Tubing	1	$49.72	$49.72	McMaster-Carr	Metal
P11	90 Degree Elbow Connector	1	$51.05	$51.05	McMaster-Carr	Metal
P12	Right-Angle Tee	1	$80.25	$80.25	McMaster-Carr	Metal
P13	High-Pressure Valve	1	$28.11	$28.11	McMaster-Carr	Metal
P14	Soft Tubing	1	$9.50	$9.50	McMaster-Carr	Polymer

## 5 Build instructions

### 5.1 Decomposition chamber

**Fig. 1:**
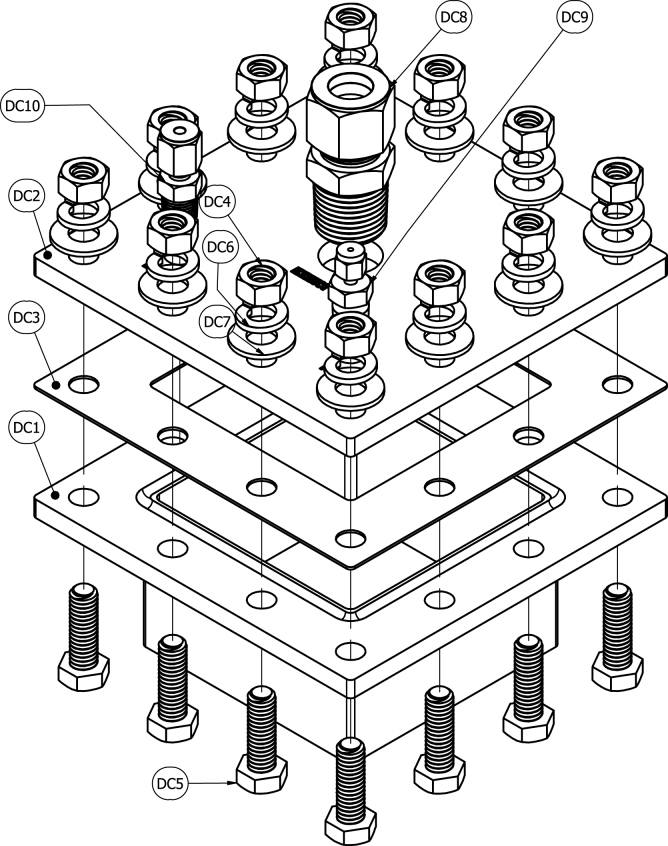
Schematic of decomposition chamber.

**Fig. 2:**
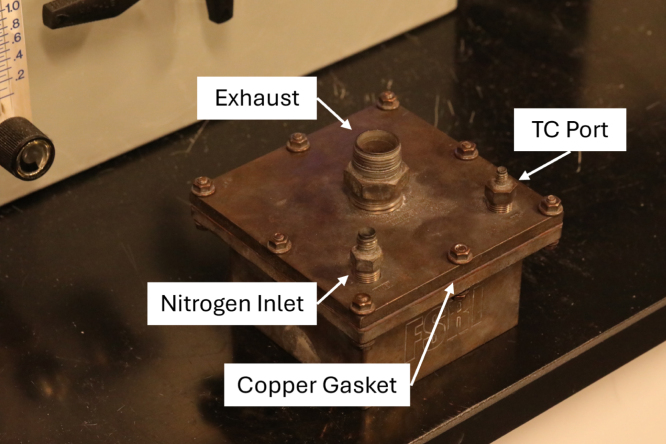
Assembled decomposition chamber.

A schematic of the decomposition chamber is shown in Fig. 1. An assembled example of the decomposition chamber is shown in Fig. 2. Three bores through the lid of the decomposition chamber (DC2) are machined with NPT threads. One of these threaded bores has 1/2 in. NPT threads through the entire depth of the bore and the other two have 1/8 in. NPT threads through the depth of the bore. A 1/2 in. tube by 1/2 in. NPT through-wall connector (DC8) is screwed into the bore with the 1/2 in. NPT threads, a 1/8 in. tube by 1/8 in. NPT through-wall connector (DC10) is screwed into one of the bores with 1/8 in. NPT threads, and a 1/16 in. tube x 1/8 in. NPT through-wall connector (DC9) is screwed into the other bore with 1/8 in. NPT threads.

A nickel-based anti-seize lubricant is applied to the threaded interface areas of all the decomposition chamber fasteners and fittings. The copper gasket (DC3) is positioned between the decomposition chamber lid (DC2) and the decomposition chamber base (DC1) such that all holes are aligned. Before each use, inspect the copper gasket (DC3) for oxidation and defects. DC3 is designed to be a consumable part. The life expectancy of DC3 is a factor of how much it is used. If the gasket sealing surfaces are cleaned and leaks are still present, it is time to replace DC3. Twelve hex-head screws (DC5) are inserted through the holes from the base to the lid. A washer (DC7) is placed around the threaded end of each screw, and a lock-washer (DC6) is placed on top of each washer. A hex-head nut (DC9) is screwed onto each screw to a torque of at least 11 ft-lbs.

### 5.2 Bubble vent

**Fig. 4:**
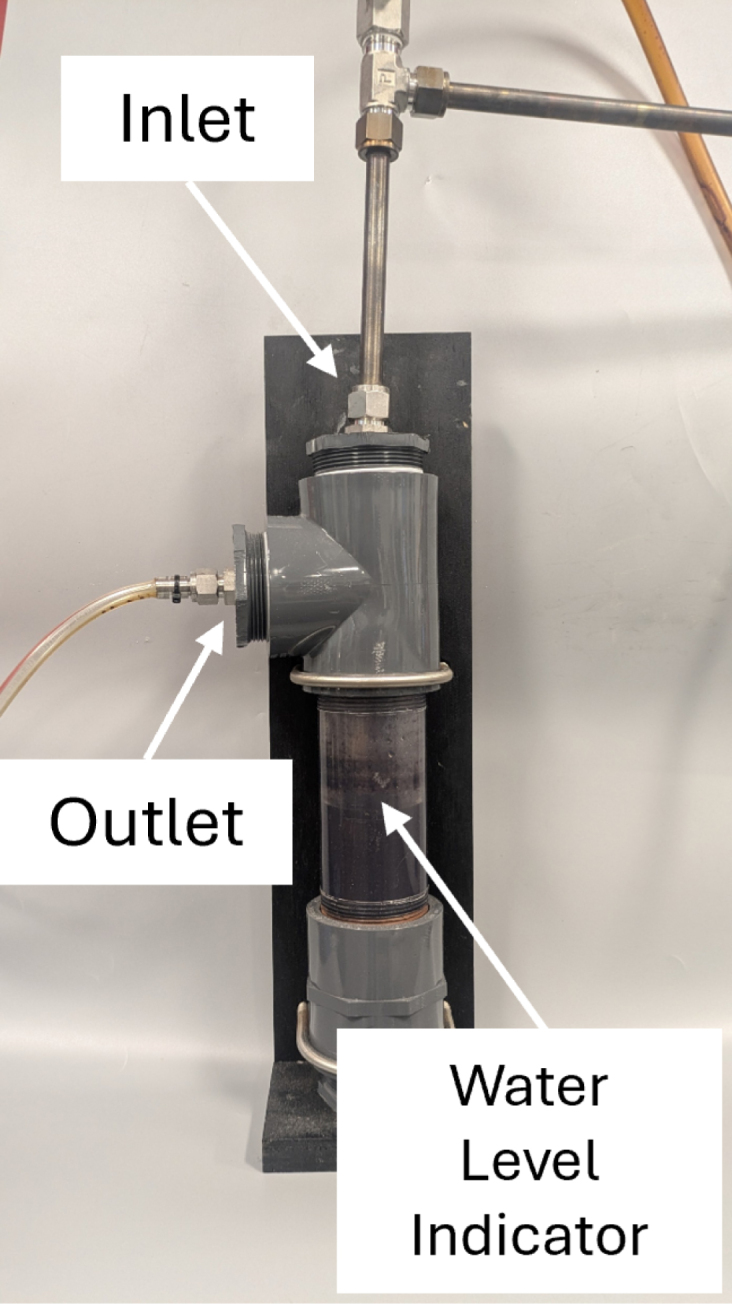
Assembled Bubble Vent.

A schematic of the bubble vent is shown in Fig. 3. An assembled example of the decomposition chamber is shown in Fig. 4. The ferrules from one end of a 1/2 in. through-wall connector (BV9) are compressed onto a 12 in. long 1/2 in. diameter tube with 1 in. gradations marked on the exterior. A 3/4 in. diameter hole is drilled through the center of a 2 in. NPT male pipe plug (BV3). The 1/2 in. through-wall connector (BV9) is connected through the drilled hole in the pipe plug with the 12 in. tube (BV11) connected to the fitting extending in the direction of the threads of the pipe plug. Silicone sealant is used to ensure an airtight seal between the through-wall fitting and the pipe plug.

The male pipe plug with the through-wall fitting and the graduated tubing is screwed into a 2 in. female NPT tee fitting (BV4). An additional 2 in. NPT male pipe adapter (BV6) is screwed into the 2 in. female NPT tee fitting (BV4) with a mesh filter (BV10) places inside the tee fitting (BV4). A 2 in. NPT male pipe plug (BV2) is screwed into a 2 in. NPT female straight connector (BV1). A transparent 6 in. long 2 in. NPT pipe nipple (BV5) is attached to the opposite end of the straight connector (BV1). The opposite end of the transparent pipe nipple is screwed into the NPT tee fitting (BV4) such that the graduated tube is concentric with the pipe nipple.

A 1/2 in. NPT to 2 in. NPT adapter (BV7) is screwed onto the 2 in. NPT male pipe adapter (BV6). A 3/8 in. tube to 1/2 in. NPT male fitting (BV8) is installed in the 1/2 in. NPT female end of the pipe adapter (BV7). Teflon thread sealant tape is used to create an airtight seal on all NPT thread connections. 3/8 in. soft PVC tubing (P14) is used to connect the BV to the SC. Soft tubing (P14) length will be determined by the proximity of the BV to the System Control Module. The bubble vent is placed in line between the decomposition chamber and oxygen sensor module to function as a flame arrestor, preventing damage to downstream components.

### 5.3 Oxygen sensor module

A schematic of the oxygen sensor module is provided in Fig. 5. Two 1/4 in. through-wall connectors (OSB3) are installed through the holes in the sides of the 3D-printed oxygen sensor module base (OSB2) with an O-ring (OSB13) between each through-wall connector and the module base on the interior side of the module base to create an airtight seal.

An 8-pin electrical receptacle (OSB7) is installed through the wall of the module base (OSB2). 14 mm long stainless steel M3 × 0.5 mm machine screws (OSB11), stainless steel washers (OSB12), and M3 × 0.5 mm nuts (OSB10) are used to secure the receptacle (OSB7) and the receptacle gasket (OSB9) to the exterior of the module base. An oxygen sensor (A2) and an environmental sensor (A5) are mounted to the underside of the oxygen sensor module lid (OSB1). The oxygen sensor (A2) is intended to be a consumable component and should be replaced in accordance with manufacture guidelines. The oxygen and environmental sensors are connected to the 8-pin electrical receptacle (OSB7) via a 8-pair cable (E23).

The oxygen sensor module lid (OSB1) is secured to the oxygen sensor module base (OSB2) by passing four 3-1/4 in. long 1/4-20 machine screws (OSB5) through the four holes through the corners of the base. A square-profile o-ring (OSB6) is installed between the base and the lid to ensure an airtight seal. Flange nuts (OSB4) are tightened onto the machine screws on the lid side of the module to generate the downward pressure necessary for the airtight seal.

### 5.4 System control module

Photographs of the system control module are provided in Fig. 8, Fig. 9. The system control module is comprised of a slant-top enclosure (SC1) with various mounting hardware, plumbing fittings, and electrical connections. Holes were drilled through the slant-top enclosure to accommodate mounting for electrical and plumbing components. Precise locations of these holes is not important and have been excluded from this description. Rectangular holes, one measuring 3-1/4 in. wide by 1 in. tall, and another measuring 3-5/16 in. wide by 3-1/8 in. tall, are cut into the slant-top enclosure to allow the thermocouple connectors (A9) and LCD Displays (A4) to be installed through the wall of the enclosure. Circular holes with a diameter of 1-1/2 in. were drilled into the slant-top enclosure to allow for installation of the cooling fan (E4) and ventilation intake (E5). The thermocouple connector assembly, the ventilation intake, and the cooling fan are attached to the enclosure with four screws (SC7), washers (SC6), and nuts (SC5). The LCD display was connected to the enclosure with four hex head screws (SC9), washers (SC8), and serrated flange lock-nuts (SC10).

**Fig. 6:**
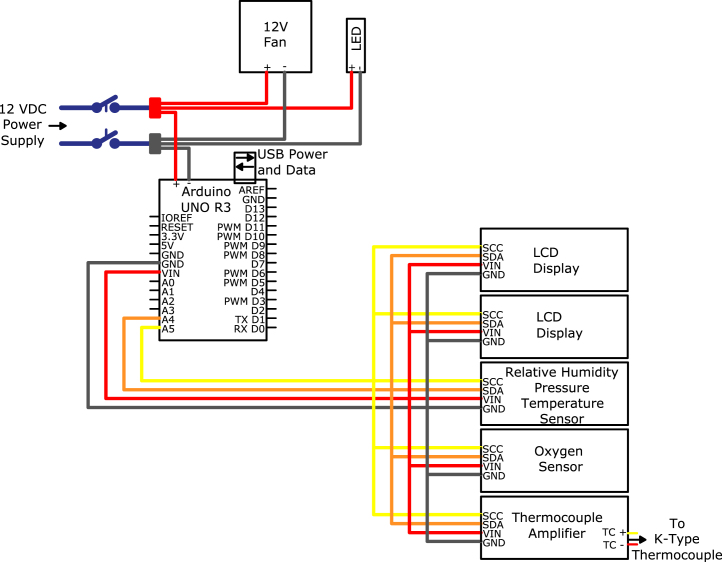
Wiring Diagram for Electrical Components in SC.

A wiring diagram which shows all connections and components in the SC is provided as Fig. 6. Each of the six thermocouple connectors in the assembly is connected to the thermocouple adapter module (A1) with type K thermocouple wire (A10). The thermocouple amplifier is connected to the Arduino (A3) with an adapter cord (E24). The Arduino is mounted to a DIN 3 rail (SC3) with a terminal block adapter (E11). A set of color-coded terminal blocks (E13, E14, E15, E16), separated by terminal block end covers (E12) and terminated on each end by end stops (E17) are mounted on a DIN 3 rail (SC3). The color-coded terminal blocks are connected to the Arduino with matching color solid core wire (E6, E7, E8, E9).

**Fig. 7:**
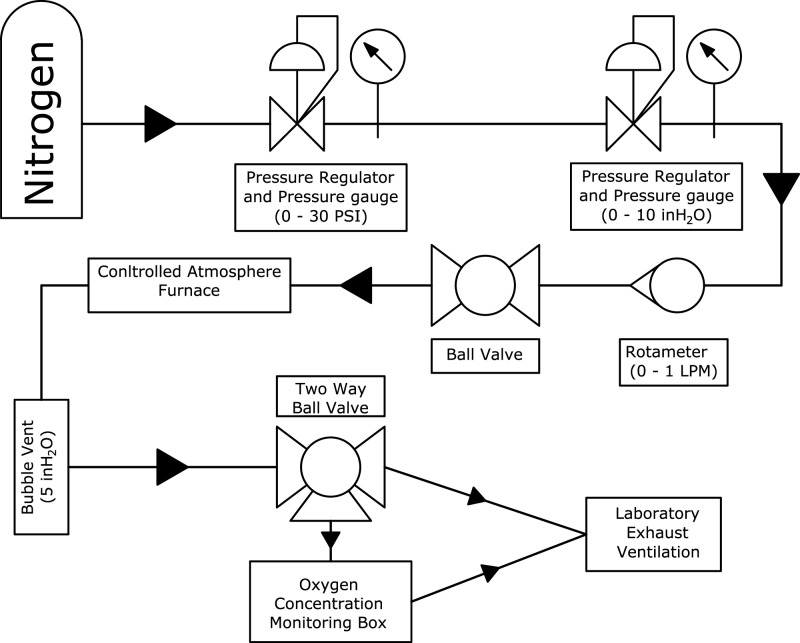
Gas flow diagram.

A gas flow diagram which shows all plumbing connections and directions of gas flow is provided as Fig. 7. Five 1/4 in. through-wall straight compression fitting connectors (P3) are installed through the enclosure. The nitrogen supply is connected to a low pressure gas regulator (P1) with two compression to NPT adapters (P2). The low pressure side of the regulator is connected to a through-wall connector (P3). The interior side of this through-wall connector is connected to a panel-mount valve (P4) via 1/4 in. flexible tubing (P7). The outlet of the panel-mount valve is connected to a 1 L/min panel-mount rotameter (P5) using two compression to NPT adapters (P2). The outlet of the rotameter is connected to a through-wall connector (P3) which directs the nitrogen flow to the DC.

The outlet of the BV is attached to smooth-bore stainless steel tubing (P8) which is bent to reduce tension on the tubing connecting the BV to the SC. The bent tubing is attached to a through-wall connector (P3) which is connected to a panel-mounted diverting valve (P6) with flexible tubing (P7). One outlet of the diverting valve is directed to the OSB, while the other is directed out of the SC via a through-wall connector (P3). The outlet of the OSB is also directed to out of the SC via a through-wall connector (P3).

### 5.5 Assembly

**Fig. 10:**
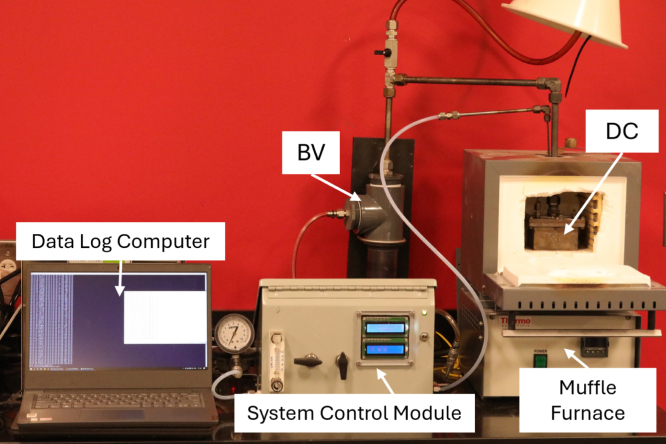
Benchtop assembly of the APC.

Fig. 10 illustrates an example of a fully assembled APC setup. The material samples are placed in the DC, and the DC is sealed as described in Section 5.1. The purge gas outlet connects to 1/4 in. smooth bore tubing (P8) which is connected to a reducing elbow (P9) with 1/8 in. smooth bore tubing (P10) which is directly connected to the lid of the DC. The elbow directs the 1/8 in. tubing downward through the furnace exhaust hole to allow for connection and purge gas flow to the DC.

A 1/2 in. smooth bore tube is connected to the exhaust on the lid of the DC to allow the products of decomposition and the purge gas to flow out of the DC. The tube is connected to an elbow compression fitting (P11) and additional 1/2 in. tube. The tube connects to a right-angle adapter (P12) that is attached to the 1/2 in. tubing in the BV (BV11). The adapter (P12) is also connected to a valve (P13) that allows the user to bypass the BV if necessary. The outlet of the BV is connected to the SC via 3/8 in. flexible tubing (P14).

A 1/16 in. diameter thermocouple (Inconel sheathed, K-type, 24 gauge, ungrounded) with a molded mini connector (A8) is inserted into the DC through the 1/16 in. tube compression fitting (DC9) on the lid to the chamber. The thermocouple is attached to the SC via duplex type-K thermocouple wire (A10). A second length of thermocouple wire is wired between the molded mini connector and the furnace control input connection to ensure that the furnace provides the necessary power to maintain the set point temperature or temperature ramp profile in the DC.

## 6 Operation instructions

The APC is intended to initiate thermal decomposition of potentially combustible materials. Because this process can generate flammable fuel vapors, there is the potential for ignition of those fuel vapors within the DC, and consequently an explosion hazard, if there is oxygen present in the DC. The APC was designed to resist the intrusion of air from the surrounding atmosphere and to mitigate this explosion risk by purging oxygen from the DC, but the operator must be aware of the possibility of an over-pressure in the system. Under the intended operating procedure, the DC is continuously purged with an inert gas to force non-oxidative thermal decomposition, eliminating the possibility of an ignitable fuel-air mixture forming in the DC. Operating instructions are included here for this procedure.

The first step in producing pyrolzed samples with the APC is to record the mass and dimensions of each sample, before sealing the samples inside the DC. The DC is then positioned in the muffle furnace. The thermocouple and tubing (which carry purge gases into and out of the DC) are routed through the port in the top of the muffle furnace and are attached to the DC. The BV is filled with water to the 5 in. level, and the nitrogen pressure regulator is adjusted to a pressure that is slightly greater than the pressure generated from the BV water level. This will allow purge gas to flow through the DC and bubble through the water inside the BV.

Purge gas flow is initiated to the decomposition chamber by adjusting the rotameter to a flow rate of 1 L/min. Flow of the purge gas through the system is verified through observation of bubbles in the bubble vent and a rapid decrease in the oxygen concentration measured in the purge gas flow. When the oxygen concentration in the purge gas is effectively zero (0 ± 0.01 vol.%), this will take approximately two minutes. Next, the furnace temperature controller is adjusted to the desired set point temperature. The temperature measured in the decomposition chamber is monitored to ensure that the temperature approaches the set point and the oxygen concentration in the purge gas path is monitored to ensure that no leaks are present in the decomposition chamber. The APC is allowed to reach the set point temperature and soak at that temperature for the desired soak duration. To bring the system back to room temperature, the set point temperature is decreased to room temperature and the measured temperature is monitored to ensure proper operation.

## 7 Validation and characterization

The use case for which the APC was initially developed involved production of structurally stable thermally decomposed material samples to allow for measurement of the thermal diffusivity of the produced samples through a range of environmental temperatures [1]. The APC was used to control the environment at an elevated temperature in a nitrogen atmosphere to produce the thermally decomposed samples. The initial plan was for the samples to be tested over the temperature range that the material would be expected to experience in a fire environment using transient plane source [4], modified transient plane source [5], and laser flash [6] apparatuses to measure the thermal diffusivity. Due to challenges encountered measuring thermal diffusivity with the transient plane source and modified transient plane source at elevated temperatures, only the laser flash apparatus was used to characterize the sample. This sample preparation process was performed for a eucalyptus hardboard to validate the capabilities of the APC.

The thermocouple installed in the APC was connected to the temperature controller such that the APC temperature was the controlled variable. Characterization of the instrument required an understanding of the thermal inertia and rise time of the APC temperature as well as characterization of the ability of the device to fully purge oxygen from the DC. Validation of the instrument required validation of the temperature and oxygen concentration of the environment generated within the chamber. The oxygen measurement was validated by flowing nitrogen through the system and monitoring the decrease in oxygen concentration as the system was purged of air. A soap solution was applied at all plumbing fittings to ensure no leaks were present while nitrogen flowed through the system.

**Fig. 11:**
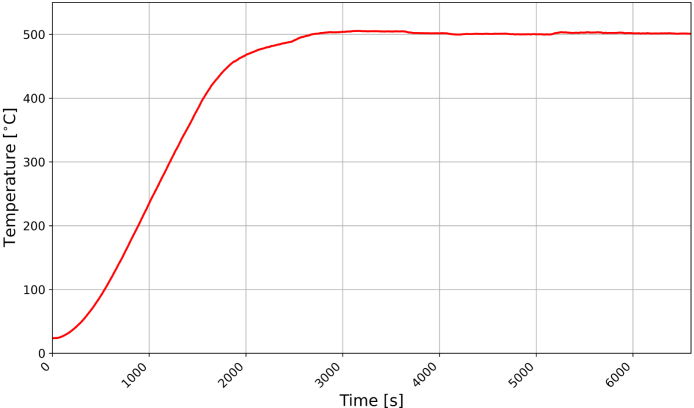
Example of Temperature History of APC for Eucalyptus Hardboard Decomposition.

To prepare the eucalyptus hardboard samples with the APC, the chamber was heated to 500 °C at a rate of approximately 16 °C/min and held at 500 °C for 90 min while purging the chamber with ultra high purity nitrogen at a rate of 0.9 L/min. While any heating program may be applied, in this case it was desired to increase the temperature linearly at this rate. A plot of the chamber temperature measured during the ramp up and soaking phases of the thermal decomposition process are provided in Fig. 11.

The soak temperature and duration were determined based on isothermal thermogravimetric analysis (TGA) tests [7]. The TGA data were collected on a eucalyptus hardboard prepared as a powder through cryogenic grinding. TGA tests were conducted with a heating rate of 30 °C/min up to the set point temperature and held at the set point temperature for various lengths of time to establish a relationship between soak time and residual mass. The fraction of the initial mass remaining at the time where mass loss became negligible in TGA tests served as a benchmark for the performance of the APC. The TGA residual mass at 500 °C after one hour was 22.0 ± 0.2% and the APC residual mass at 500 °C after one hour was 25 ± 4%. The results of these experiments indicated that the APC was able to decompose the eucalyptus hardboard to a state that was consistent with the residue remaining after a thermal analysis experiment to allow for additional experimentation that required larger-scale sample specimens.

An additional study which used the APC to prepare samples for further measurement was conducted to investigate the evolution of thermal conductivity with exposure temperature and exposure duration for char formed from densified wood samples [8]. In this work, the flash method [6] was used to measure the thermal diffusivity of the partially decomposed samples. The flash method directly measures heat flow through the sample, so accurate measurement requires a thermally stable sample, i.e. a sample that does not continue to decompose at a given set point temperature.

In this work, the temperature of the APC was increased along a prescribed temperature ramp of 1 °C/min up to a maximum set point temperature of 500 °C. Samples prepared according to this protocol were tested in dynamic thermogravimetric experiments in nitrogen to verify thermal stability and it was found that the samples lost approximately 2% of their initial masses up to 500 °C, which effectively confirmed the samples prepared in the APC would have sufficient thermal stability to be reliably tested according to the flash method. The study revealed the importance of the temperature history of a char sample on its thermal transport properties.

## CRediT authorship contribution statement

**Ryan T. Chaffer:** Writing – original draft, Investigation, Conceptualization. **Joseph Johnson:** Methodology, Conceptualization. **Matthew J. DiDomizio:** Writing – review & editing, Supervision, Conceptualization. **Mark B. McKinnon:** Writing – original draft, Supervision, Project administration.

## Declaration of competing interest

The authors declare that they have no known competing financial interests or personal relationships that could have appeared to influence the work reported in this paper.

## References

[1] DiDomizio Matthew J., McKinnon Mark B., Bellamy Grayson (2024). Measurement of thermal conductivity of thermally reactive materials for use in pyrolysis models. Fire Mater..

[2] Quaresma Tássia L.S., Hehnen Tristan, Arnold Lukas (2024). Sensitivity analysis for an effective transfer of estimated material properties from cone calorimeter to horizontal flame spread simulations. Fire Saf. J..

[3] Chaffer Ryan, McKinnon Mark (2023).

[4] (2015).

[5] (2016).

[6] (2022).

[7] (2017).

[8] Bellamy G.T., McKinnon M.B. (2024). Methodology for the determination of the thermo-physical properties of densified wood over thermal decomposition. J. Phys.: Conf. Ser..

